# Editorial: Considerations in the surgical management of HPV-positive oropharyngeal squamous cell carcinoma

**DOI:** 10.3389/fonc.2023.1205525

**Published:** 2023-06-13

**Authors:** Jon Mallen-St Clair, Ryan K. Orosco

**Affiliations:** ^1^ Samuel Oschin Comprehensive Cancer Institute, Cedars-Sinai Medical Center, Los Angeles, CA, United States; ^2^ Department of Surgery, Division of Otolaryngology-Head and Neck Surgery, Cedars-Sinai Medical Center, Los Angeles, CA, United States; ^3^ Department of Surgery, Division of Otolaryngology-Head and Neck Surgery, University of New Mexico, Albuquerque, NM, United States; ^4^ University of New Mexico Comprehensive Cancer Center, Albuquerque, NM, United States

**Keywords:** transoral robotic surgery (TORS), transoral robotic surgery (TORS) for oropharynx cancer, HPV oropharyngeal squamous cell carcinoma (OPSCC), unknown primary cancer, clinical trial, surgical oncology, radiation oncology

Over the past two decades, we have become quite familiar with the epidemic of human papillomavirus related oropharyngeal squamous cell carcinoma (“HPV-positive” or “HPV-mediated” OPSCC). HPV-positive OPSCC is a unique disease with a distinct set of risk factors, oncogenic pathophysiology, and natural history that distinguishes it from its HPV-negative counterpart. Importantly, HPV-positive OPSCC carries a better prognosis and favorable response to therapy.

This novel clinical entity begs a number of questions. How do we detect this cancer earlier? What do we do when we don’t see a primary tumor? How can we best support our patients through their treatment? What is the optimal treatment? Can we do “less” treatment? How do we know which patients are candidates for less-aggressive treatment? How much radiation is “enough”? Does a surgery-based or radiation-based treatment plan provide better functional and oncologic outcomes? What novel agents are better than our current therapies? Are there key associations or modifiable risk-factors that we are missing? What can we do to prevent this cancer all together? How do we optimize the value of our cancer care? These are some of the topics that are touched upon in this Special Edition of Frontiers in Oncology - section Surgical Oncology. Herein, we compile and present a collection of manuscripts that stem from research and review efforts spanning Europe, Asian, and North America. These manuscripts offer a contemporary perspective on several of the key questions in our field.

HPV-mediated oncogenesis relies on the HPV genome and its major oncogenes E6 and E7. Chitsike et al. present us with benchtop research into a novel HPV E6 inhibitor (GA-OH) as a potential radiosensitizing agent. Their work with head and neck cancer cell lines demonstrated differential effects between HPV-positive and HPV-negative cancer cells. GA-OH performed better than cetuximab, but not as well as cisplatin. We look forward to subsequent work evaluating viral oncogene inhibitors to add to our armamentarium of treatment options.

In 2023, cancer treatment is not one size fits all. With our increased scientific understanding of the pathogenesis of oropharyngeal cancer, we can now risk stratify patients at a molecular level. Given the improved outcomes of patients with HPV-positive OPSCC, multiple clinical trials are evaluating treatment de-intensification to preserve swallowing function and reduce the morbidity of treatment while maintaining a high rate of cure. Lin et al. and Mensour et al. provide an overview of the current state of surgical trials and our latest efforts in treatment de-intensification. We benefit from results from the early trials in this arena, and look toward the results from ongoing and up-and-coming clinical trials.

Given the morbidity of the surgery required to gain access to the oropharynx, chemoradiation dominated the treatment of oropharyngeal cancer prior to the advent of minimally invasive approaches using transoral surgery. Since the development of transoral laser microsurgery (TLM) and the birth of transoral robotic surgery (TORS), we have traveled quite far down the minimally invasive surgery road. TORS gained traction in our field by playing a crucial role to enable safe and effective treatment of oropharynx cancer while preserving quality of life. FDA approval of the da Vinci surgical robot (Intuitive Surgical Inc., Sunnyvale, CA) for transoral otolaryngologic procedures in 2009. Early studies demonstrated excellent functional outcomes, with decreased need for feeding tubes, improved quality of life and oncologic soundness. The da Vinci robot has allowed the development of less morbid surgical procedure in treating oropharyngeal cancer which had previously relied on lip split mandibulotomy with tracheostomy and gastrostomy tube placement. In the modern era these surgical procedures can be done typically without even temporary nasogastric feeding. As a field, we have learned a great deal about how to utilize and when to employ this robotic surgery tool to optimize patient outcomes.

Kalavacherla et al. provides us with a review of the management of the unknown primary head and neck cancer. We are guided along the diagnostic workup for these patients and see the critical role that TORS can play in searching for the primary tumor deep within the crowded and cryptic confines of the oropharynx. Further innovations have brought successive generations of da Vinci models and the latest iteration – the single port SP is currently under evaluation with favorable initial outcomes (1). In the manuscript by Chai et al. from the Icahn School of Medicine at Mount Sinai, we see a new approach to utilizing TORS in the context of a novel risk-stratification schema based on a biomarker—circulating tumor DNA. These are exactly the types of clinical trials that will continue to push our field forward and help clarify the optimal treatment intensity for each individual patient.

Kanzow et al. bring us a unique perspective by looking at OPSCC through the dentistry and oral surgery lens. They looked at outcomes for patients with HPV-positive and HPV-negative OPSCC to see if there were associations, they could identify related to oral health. In their cohort of 119 patients, they did not detect a significant signal for oral health, but we applaud their fastidious work that certainly merits ongoing study. This gets to the questions about key associations, modifiable risk factors, and multi-faceted care for our patients.

Finally, we hear from Guo et al. regarding the management of recurrent HPV-mediated OPSCC. The prognosis of HPV-positive OPSCC can be described as “favorable” compared to HPV-negative disease, but cancer cure is by no means guaranteed. About a quarter of patients will experience recurrence or progression 3-years out from primary therapy. Surgical salvage has a different paradigm for HPV-mediated OPSCC, and it is important to delve into this topic and recognize the subtleties and the evidence that supports our practices. For example, in the setting of lung metastases, surgical salvage *via* “metastatectomy” can be considered. This approach turns conventional practices (for HPV-negative disease) on their head, but have a sound rationale and data to support. Re-irradiation is another complex topic in and of itself, that we are oftentimes forced to confront with recurrent disease. Furthermore, recurrent disease challenges us to evaluate the potential for second-line systemic therapy and novel immunotherapy regimens. Understanding the latest innovations of our literature will help us navigate these challenging considerations.

We hope that you find this special edition of Frontiers in Oncology to be informative, thought provoking, and beneficial to your patients. At our current state of the art, we do not know the best way to evaluate, risk stratify, treat, support, and follow our patients with HPV-positive OPSCC. We can only continue to strive for continued improvement and innovation in these realms. We are encouraged that continued research will enlighten us on these issues, With any luck within the decade a second special edition will allow further clarity on the optimal treatment for these patients.

## Author contributions

All authors listed have made a substantial, direct, and intellectual contribution to the work and approved it for publication.

## References

[1] Van Abel K Yin L Price D Janus JR Kasperbauer JL Moore EJ. One-year outcomes for da Vinci single port robot for transoral robotic surgery. Head Neck (2020) 42(8):2077–87. 10.1002/hed.26143 32190942

